# APT mass spectrometry and SEM data for CdTe solar cells^☆^

**DOI:** 10.1016/j.dib.2016.03.042

**Published:** 2016-03-16

**Authors:** Jonathan D. Poplawsky, Chen Li, Naba R. Paudel, Wei Guo, Yanfa Yan, Stephen J. Pennycook

**Affiliations:** a Center for Nanophase Materials Sciences, Oak Ridge National Laboratory, Oak Ridge, TN 37831, USA; b Department of Lithospheric Research, University of Vienna, Vienna, Austria; c The University of Toledo, Department of Physics and Astronomy, McMaster Hall, 2nd Floor Rm 2017, Toledo, OH 43606, USA; d National University of Singapore, Department of Materials Science and Engineering, Singapore

**Keywords:** Scanning electron microscopy, Atom probe tomography, Mass spectroscopy, Solar cells

## Abstract

Atom probe tomography (APT) data acquired from a CAMECA LEAP 4000 XHR for the CdS/CdTe interface for a non-CdCl_2_ treated CdTe solar cell as well as the mass spectrum of an APT data set including a GB in a CdCl_2_-treated CdTe solar cell are presented. Scanning electron microscopy (SEM) data showing the evolution of sample preparation for APT and scanning transmission electron microscopy (STEM) electron beam induced current (EBIC) are also presented. These data show mass spectrometry peak decomposition of Cu and Te within an APT dataset, the CdS/CdTe interface of an untreated CdTe solar cell, preparation of APT needles from the CdS/CdTe interface in superstrate grown CdTe solar cells, and the preparation of a cross-sectional STEM EBIC sample.

**Specifications table**

**Table:** 

Subject area	*Materials Science*
More specific subject area	*Solar Cells, CdTe, STEM, SEM, atom probe tomorgraphy (APT), EBIC*
Type of data	*SEM secondary electron images, mass spectra, 3D atomic positions*
How data was acquired	*Hitachi S4800 SEM, FEI Nova 200 dual beam FIB, CAMECA LEAP 4000 XHR,*
Data format	*Images, graphs, tables*
Experimental factors	*The APT measurments were performed in laser mode with 30 pJ, 355 nm,~10 ps, 100 kHz laser pulses at a 30 K base temperature. The SEM-SE images shown in*Fig. 3*were acquired using a Hitachi S4800 SEM equipped with a cold field emission electron gun. The SEM-SE data shown in*Fig. 4*was acquired using a FEI Nova 200 dual beam FIB with a 5 kV accelerating voltage and ~1 nA of beam current.*
Experimental features	*Standard cross-sectional TEM preparation, APT was performed in laser mode with a 3 pJ laser energy, contacts were applied via FIB Tungsten*
Data source location	*Oak Ridge National Laboratory, Oak Ridge, TN*
Data accessibility	*Data are presented in this article*

**Value of the data**

• The SEM data for the cross-sectional STEM EBIC sample preparation evolution can be applied to several other material systems for others to perform STEM-EBIC measurements.
• The SEM data for the APT needle preparation evolution of the CdS/CdTe interface in superstrate grown CdTe solar cells can be used to prepare CdS/CdTe interfaces for APT analysis.
• The mass spectroscopy data reveals peak overlaps between Te and Cu. These peaks need to be deconvoluted to obtain the appropriate Te and Cu compositions.
• The APT data reveals significant S diffusion across the CdS/CdTe interface without a CdCl_2_ heat-treatment.

## 1 Data

**Fig. 1:**
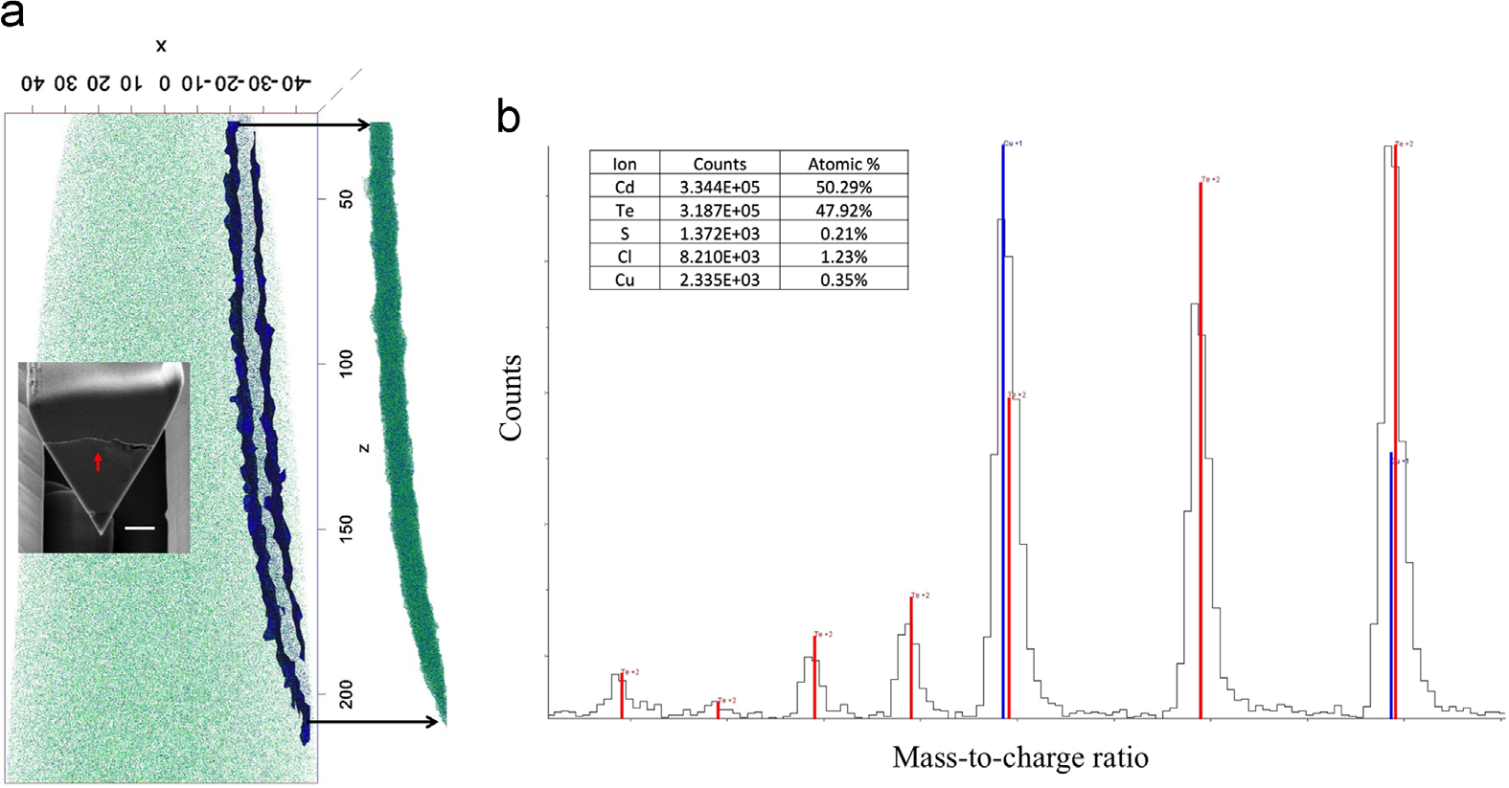
(a) The APT reconstruction of a CdTe grain boundary <500 nm from the back contact of the device including the approximate location of the GB in the inset with a 1 µm scale bar (white). A 1% Cl and S isosurface was used to extract the GB region from the dataset shown to the right of the reconstructed needle. (b) The mass spectrum of the extracted GB region including the expected Te^++^(red lines) and Cu^+^ (blue lines) peak ratios from the isotopic abundances. The inset shows the ion concentrations within the extracted GB region after the manual peak decomposition.

The data presented in this article is comprised of a series of SEM images, a CdTe based mass spectrum, and an APT dataset. The dataset in Fig. 1 is a mass spectrum collected from a CdTe solar cell APT dataset with a GB enclosed in the APT needle. The mass spectrum range displayed shows the eight isotopes of the Te^2+^ ion, which include mass-to-charge state ratios of 59.95. 60.95, 61.45, 61.95, 62.45, 62.95, 63.95, and 64.96 Da. The Te^2+^ mass-to-charge ratios of 62.95 and 64.96 Da overlap with Cu^+^ mass-to-charge state ratios of 62.93 and 64.93 Da because the mass resolving power of the LEAP 4000 XHR atom probe is not sufficient to separate these peaks (~1000).

**Fig. 2:**
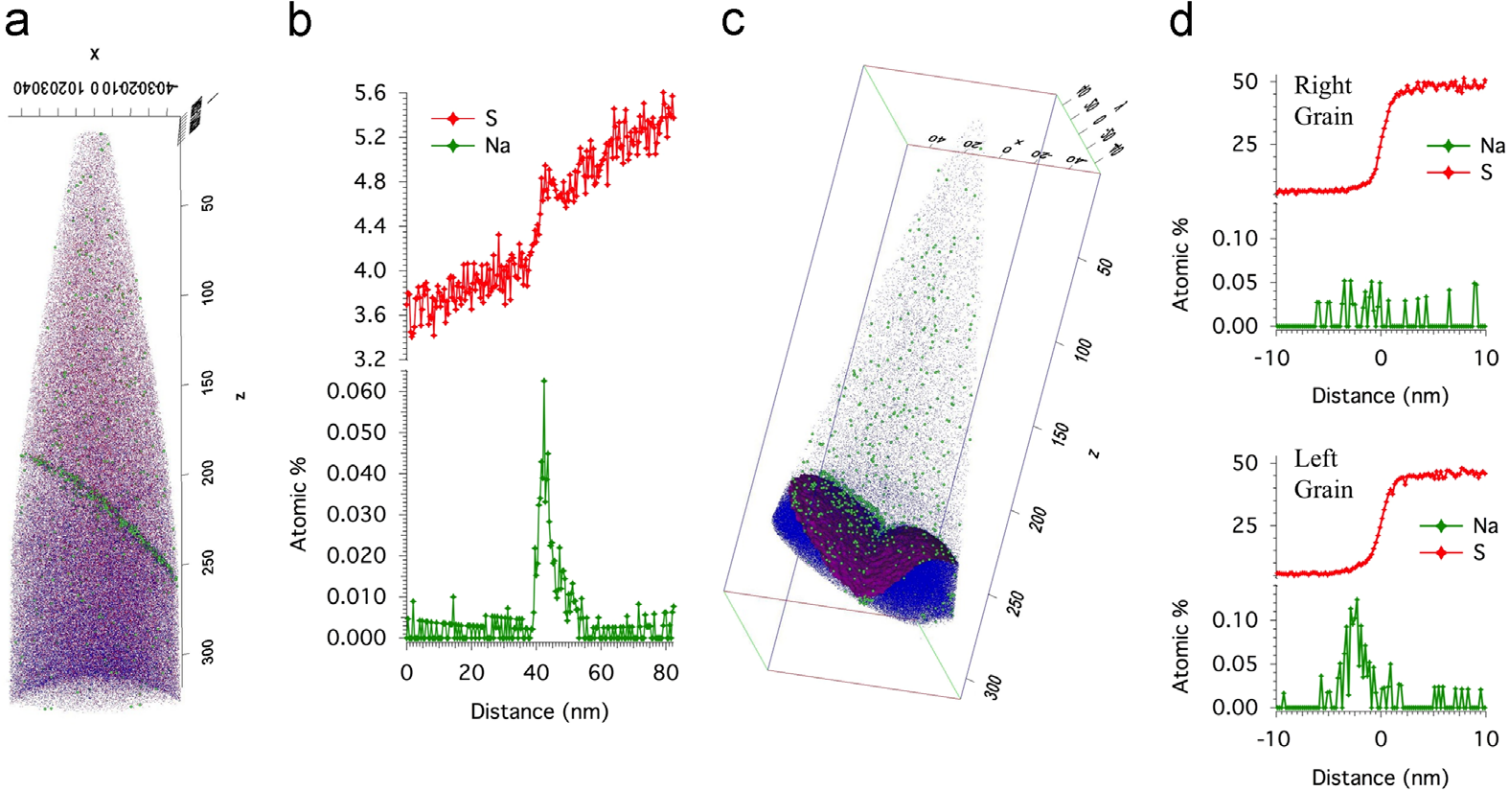
APT data for an untreated CSS grown CdTe superstrate device. (a) A reconstructed APT needle of a GB ~60 nm from the CdS/CdTe interface. The needle fractured close to the CdS/CdTe interface. (b) The S and Na concentrations for a 1D line profile across the GB shown in (a). (c) A reconstructed APT needle of the CdS/CdTe interface with the S (blue) and Na (green) ions displayed. Also, a magenta 22% S isoconcentration surface clearly shows the CdS/CdTe interface with a CdS GB. (d) Proximity histograms of the 22% S isoconcentration surface for each isolated grain.

The datasets presented in Fig. 2(a) and (c) are 3D APT reconstructed volumes displaying the atomic positions of Te (red), S (blue), and Na (green) atoms as small colored dots. The displayed scale bars are in nms. The data presented in Fig. 2(b) is the resulting compositional gradient from a 1D line profile across the grain boundary shown in Fig. 2(a). Fig. 2(d) shows the data resulting from proximity histograms of the 22 at% S isosurface shown in Fig. 2(c). Each grain shown in Fig. 2(c) was isolated to generate the proximity histograms.

**Fig. 3:**
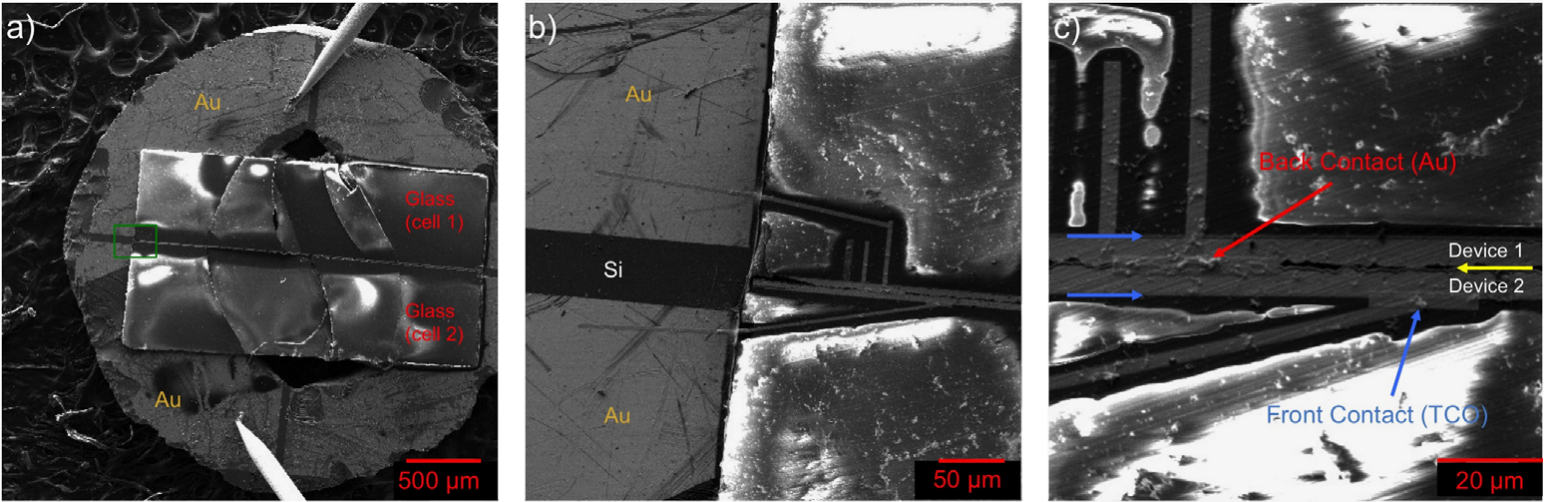
(a) A SEM image of a mechanically polished TEM cross-section “sandwich” mounted on a special electrical contact 3 mm grid with 4 isolated gold contact pads. The sample shown in the picture is broken and was not used for the measurement. (b) A higher magnification image of the region highlighted by the green box in (a). FIB Pt contacts were deposited from the Au pad to the CdTe device. (c) An SEM image of the contacted device. STEM EBIC is only possible for device 2 in this configuration because the FIB Pt for the back contact shorts device 1. The blue arrows indicate the ITO layers, and the yellow arrow is the position of the Au back contact for each device.

**Fig. 4:**
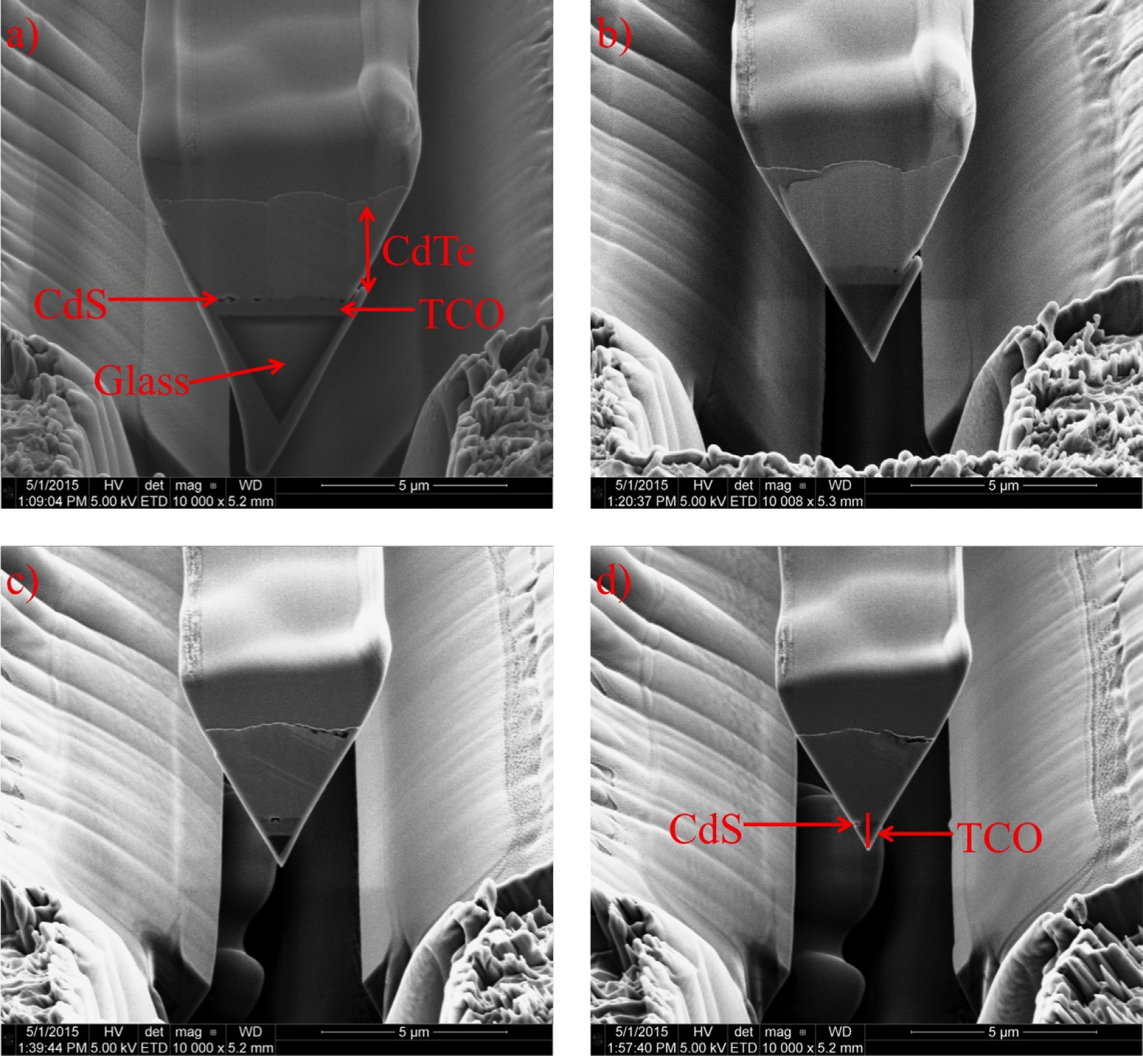
SE images of a CdTe wedge cut for in-situ lift out of the CdS/CdTe interface for APT needle preparation. (a) The initial wedge cut showing the CdTe, CdS, TCO, and glass layers. (b)–(d) Subsequent 22 degree stage tilt FIB cuts to remove the glass layer and preserve the CdS/CdTe interface. The red line in (d) indicates the APT needle position after the 5 kV final milling step.

Several SEM-SE datasets are displayed as images in Fig. 3, Fig. 4. Fig. 3 contains three SEM-SE images that show a polished cross-sectional STEM sample mounted on a 3 mm grid with 4 gold contacts obtained at three different magnifications. Fig. 4 shows four SEM-SE images of a wedge cut CdTe solar cell acquired after focused ion beam (FIB) milling of the material. Each image is shown at the same magnification, and the image order (a–d) follows in a chronological order.

## 2 Experimental design, materials and methods

### 2.1 Peak decomposition in IVAS

Fig. 1 shows (a) a reconstructed CdTe needle containing a GB <500 nm from the back contact and (b) the mass spectrum associated with the extracted GB region including the expected peak ratios with respect to the isotopic abundances. It is clear from the mass spectrum that the ^63^Cu^+^ and ^65^Cu^+^ are overlapping with the ^126^Te^++^ and ^130^Te^++^ peaks. There was no indication of the ^63^Cu^+^ and ^65^Cu^+^ peaks overlapping with the ^126^Te^++^ and ^130^Te^++^ peaks for CdTe grains, GBs close to the CdS/CdTe interface, and the CdS/CdTe interface, and therefore, Cu was unable to be detected in these regions within the sensitivity of APT. The six non-overlapping Te peaks were used to estimate the number of Te counts that should occur for the ^126^Te^++^ and ^130^Te^++^ peaks. These counts were subtracted from the total counts occurring for the 63 and 65 Da peaks to obtain the number of Cu counts. The resulting composition can be seen in the inset of Fig. 1(b), which consists of all the ions contained within the 0.1 at% S and Cl isosurface shown in Fig. 1(a). This large region (~8 nm wide), which contains the entire GB, was used to increase the number of atoms, and therefore, increase the statistics for obtaining an accurate Cu composition after the peak deconvolution. A Cu concentration GB line profile could not be determined because of the need for a large number of collected ions to determine the appropriate Te ion counts in the 63 and 65 Da mass peaks. The Cu/Cl ratio in the GB was calculated to be 0.29 after peak deconvolution, and therefore, the peak Cu concentration for a GB profile with the same full width half maximum as a Cl concentration profile can be multiplied by this factor to identify the approximate maximum Cu concentration in the GB.

### 2.2 No treatment APT data

Untreated CdTe needles were run in the atom probe to understand Na and S diffusion prior to either treatment and to confirm that the segregation of Cu and Cl in GBs and interfaces does not occur for untreated cells. Possible Cu or Cl contamination in the CdTe growth chamber, subsequent annealing chambers, or in the source materials could enable for these elements to be present in an untreated sample. Fig. 2 shows (a) a reconstructed CdTe GB close the CdS interface, (b) a 1D line profile across the GB, (c) a reconstructed CdS/CdTe interface with two CdS grains, and (d) proximity histograms of the 22% S isoconcentration surface of each isolated grain. The results show that there is a similar concentration of S segregation to the GB, S concentration in the CdTe grains close to the interface, and Na segregation in the GBs compared to a fully treated sample [1]. Therefore, significant Na segregation and S diffusion occurs during the CdTe growth for CSS grown CdTe solar cells. In addition, the mass spectrum associated with these datasets did not show peaks related to Cu or Cl, and therefore, Cu and Cl segregation was not detectable using APT. It has been shown that untreated CdTe solar cells do not perform well. [2] Thererfore, S diffusion into the CdTe layer and Na segregation within GBs and the CdS/CdTe interface do not drastically improve the device performance compared to the CdCl_2_ and Cu treatments.

### 2.3 STEM EBIC sample preparation

The data presented in Fig. 3 show an experimental setup and sample preparation procedure designed to measure STEM EBIC currents from the cross-section of a solar cell. The standard argon ion mill cross-section TEM sample preparation procedure was followed to produce an electron transparent cross-section with a few exceptions. First, the CdTe devices were glued on top of each other to form a “sandwich.” The CdTe sandwich was cut as thin as possible, polished on one side, and then glued to a special 3 mm Si grid purchased from TEMwindows.com containing 4 gold contact quadrants as shown in Fig. 3(a). The CdTe region was positioned such that it was placed on the non-conducting Si region to avoid device shorting. The remaining sample was then polished to a thickness of ~10 µm, and finally thinned in a Fischione 1050 argon ion mill at liquid nitrogen temperatures until the CdTe region was electron transparent. Contacts from the CdTe device were made to the appropriate Au pads on the TEM grid. This task is specifically challenging because the n and p type CdTe contacts are separated by <4 µm in the cross-section configuration. A Hitachi dual beam FIB was used to deposit W contacts from the solar cell to the Au TEM grid contacts as shown in the SEM-SE data in Fig. 3(b) and (c). After these contacts were made, a FIB cut was made between the p and n contact of the CdTe solar cell to reduce shorting from the W spray during the FIB W deposition. The grid was then mounted on a NION electric contact cartridge in which the Au contacts of the gird were electrically connected to the cartridge using high conductive Ag paint. After the sample was inserted into the microscope, the EBIC signal was collected and amplified with a Stanford Research Instruments SR570 current preamplifier. The amplified signal was then connected to a Gatan Digiscan 2 to digitize the EBIC signal and sync it with the electron beam position.

### 2.4 APT sample preparation for the CdS/CdTe interface

The data presented in Fig. 4 show the evolution of a CdTe solar cell wedge liftout procedure with the intentions of targeting the CdS/CdTe interface within an APT needle. Successful APT runs of the CdS/CdTe interface from superstrate grown CdTe devices is a difficult task due to the close proximity of the CdS/CdTe interface (~500 nm) to the glass substrate. In order to obtain a good run with sharp time-of-flight mass spectrum peaks, there needs to be a direct thermal and electrical contact of the TCO layer to the Si micropost without a barrier of glass separating the two materials. With excessive glass underneath the finished needle, DC heating leads to non-even evaporation and unacceptably low resolution m/q peaks, in which the individual isotopes can not be separated. As shown in Fig. 4, the standard wedge lift out technique was used to remove material from the sample to be shaped into a needle on a Si microtip array [3]. Fig. 4(a) shows the first wedge cut with the 4 µm CdTe, 100 nm CdS, 400 nm TCO, and bulk glass layers identified. In order to remove the glass, FIB cuts at 22-degree tilts were made until only the ITO layer was left at the bottom of the wedge. The SEM-SE data showing the wedge shape after each subsequent cut is shown in chronological order in Fig. 4(a–d), with the final wedge shown in Fig. 4(d). The final wedge wedge was lifted out, several pieces were mounted on a Si microtip array, and each piece was sharpened. A final 5 kV milling step can be used to position the APEX of the needle 200–300 nm from the CdS/CdTe interface.
