# Supplementary material for: APT mass spectrometry and SEM data for CdTe solar cells
Source: Data Brief. 2016 Mar 16;7:779–85. doi: 10.1016/j.dib.2016.03.042 (PMC5540671; doi:10.1016/j.dib.2016.03.042)
Supplement: Supplementary file 1 — Supplementary material [file mmc1.pdf]

Conflicts of Interest Statement

Manuscript title: APT Mass Spectrometry and SEM data for CdTe Solar Cells

The authors whose names are listed immediately below certify that they have NO affiliations with or involvement in any organization or entity with any financial interest (such as honoraria; educational grants; participation in speakers' bureaus; membership, employment, consultancies, stock ownership, or other equity interest; and expert testimony or patent-licensing arrangements), or non-financial interest (such as personal or professional relationships, affiliations, knowledge or beliefs) in the subject matter or materials discussed in this manuscript.

Author names:

Jonathan D. Poplawsky, Chen Li, Naba R. Paudel, Wei Guo, Yanfa Yan, and Stephen J. Pennycook

The authors whose names are listed immediately below report the following details of affiliation or involvement in an organization or entity with a financial or non-financial interest in the subject matter or materials discussed in this manuscript. Please specify the nature of the conflict on a separate sheet of paper if the space below is inadequate.

Author names:

This statement is signed by all the authors to indicate agreement that the above information is true and correct (*a photocopy of this form may be used if there are more than 10 authors*):

Author's name (typed)	Author's signature	Date
Jonathan Poplawsky	[illegible]	03/02/2016
Chen Li		
Naba R. Paudel		
Wei Guo	Wei Guo	03/02/2016
Yanfa Yan		
Stephen J. Pennycook		

This statement is signed by all the authors to indicate agreement that the above information is true and correct (*a photocopy of this form may be used if there are more than 10 authors*):

Author's name (typed)	Author's signature	Date
Jonathan Poplawsky		
Chen Li		
Naba R. Paudel		3/01/2016
Wei Guo		
Yanfa Yan		
Stephen J. Pennycook		

This statement is signed by all the authors to indicate agreement that the above information is true and correct (*a photocopy of this form may be used if there are more than 10 authors*):

Author's name (typed)	Author's signature	Date
Jonathan Poplawsky		
Chen Li	Chen Li	20160301
Naba R. Paudel		
Wei Guo		
Yanfa Yan		
Stephen J. Pennycook		

This statement is signed by all the authors to indicate agreement that the above information is true and correct (*a photocopy of this form may be used if there are more than 10 authors*):

Author's name (typed)	Author's signature	Date
Jonathan Poplawsky		
Chen Li		
Naba R. Paudel		
Wei Guo		
Yanfa Yan		
Stephen J. Pennycook	[illegible]	2-28-16

This statement is signed by all the authors to indicate agreement that the above information is true and correct (*a photocopy of this form may be used if there are more than 10 authors*):

Author's name (typed)	Author's signature	Date
Jonathan Poplawsky		
Chen Li		
Naba R. Paudel		
Wei Guo		
Yanfa Yan	[illegible]	3/1/2016
Stephen J. Pennycook		
